# Distinct neural correlates for attention lapses in patients with schizophrenia and healthy participants

**DOI:** 10.3389/fnhum.2015.00502

**Published:** 2015-10-06

**Authors:** Ryan C. Phillips, Taylor Salo, Cameron S. Carter

**Affiliations:** Translational Cognitive and Affective Neuroscience Lab, UC Davis Center for Neuroscience, University of California, DavisDavis, CA, USA

**Keywords:** attention lapses, schizophrenia, default mode network, mind wandering, fMRI

## Abstract

Momentary lapses in attention are common in healthy populations. This phenomenon has recently received increased investigation, particularly in relationship to the default mode network (DMN). Previous research has suggested that these lapses may be due to intrusive task-irrelevant thoughts. The study of this phenomenon in schizophrenia, which is characterized by a wide variety of cognitive deficits including deficits in attention, has not previously been explored. We used the AX Continuous Performance Task to investigate attention lapses in healthy participants as well as patients with schizophrenia. We found distinct patterns of network activation between these two groups. Lapses in healthy participants were associated with DMN activation, while in patients, the same behavioral phenomenon was associated with deactivations in frontal-parietal control network (FPCN) regions. When considered in contrast to the results observed in healthy participants, these results suggest an additional origin of attention lapses in patients derived from a loss of task-related context, rather than intrusive task-irrelevant thoughts.

Maintaining attention is a challenging task, even for healthy, well-rested participants. Lapses in attention have been the subject of increasing scientific investigation (Smallwood and Schooler, 2006; Dixon et al., 2014). As attention lapses are very brief, they are generally studied indirectly. The most common methods include examining error trials in sustained attention tasks (Li et al., 2007), and modeling reaction times in order to identify reaction times that occur outside of the expected range of response times when the participant is attending (Weissman et al., 2006). Using these methods, researchers have been able to infer when attention lapses occur, and compare brain activity at these moments to moments of sustained attention.

Neuroimaging results from these studies have identified increased BOLD signal during attention lapsing in regions known to comprise the “default mode network” (DMN), as originally defined in Raichle et al. (2001). This network includes the ventromedial prefrontal cortex (vmPFC), posterior cingulate cortex (PCC), and inferior parietal lobule (IPL). While it is clear these regions are functionally connected, the functions of this network are a matter of considerable debate (Greicius et al., 2003; Andrews-Hanna et al., 2014). The DMN was originally defined by its tendency to deactivate during external attention tasks. This suggested an intrinsically oppositional relationship with the “task positive network” (Shulman et al., 1997; Fox et al., 2005). This network, which includes the dorsolateral prefrontal cortex (dlPFC), frontal eye fields (FEF), and intraparietal sulcus (IPS), increases in activation during externally focused activities. However, this anticorrelation has primarily been observed during the resting state. During task states, activation in the DMN network has primarily been associated with the engagement of specific cognitive processes such as memory retrieval (Ranganath and Ritchey, 2012), self-reflection (van der Meer et al., 2010), and theory of mind (Spreng and Grady, 2010), in addition to showing increased activity during attention lapses. Specifically, PCC activation has been associated with autobiographical memory retrieval (Ranganath and Ritchey, 2012), while vmPFC activation has been associated with self-relevance, and internally motivated, task-unrelated value (Reviewed in Andrews-Hanna, 2012). For example, vmPFC activation has reliably been observed in response to self-referent adjectives compared to adjectives describing another person. These studies suggest the possibility that attention lapses occur when task-unrelated-thoughts which involve these processes arise. In such moments, it has been suggested that attention is diverted from the external task, and toward internal “distractors” (Sonuga-Barke and Castellanos, 2007).

Schizophrenia is associated with deficits in various cognitive functions, including the control of attention (Luck and Gold, 2009) as well as reduced performance in tasks requiring vigilance (Keefe and Harvey, 2012). These deficits have been strongly associated with functional disability in the illness and are generally understood to be refractory to the widely available treatments (Green et al., 2000; Nuechterlein et al., 2011; Minzenberg and Carter, 2012). Recent work by the CNTRACS consortium has suggested that attention lapses are quite common in the illness in comparison to healthy subjects (Barch et al., 2012), and may drive the performance differences observed between healthy participants and patients in various tasks. However, attention lapses in schizophrenia have not been well characterized. One possibility is that DMN hyperactivity results in an increased rate of DMN-related attention lapses in patients with schizophrenia. Abnormalities in DMN regions have been observed in the illness, including increased activity and connectivity within this network as well as hyperconnectivity with task-positive regions (Whitfield-Gabrieli et al., 2009; Woodward et al., 2011). Specifically, previous researchers observed increased functional connectivity between the vmPFC and the dlPFC. Understanding attention lapsing in schizophrenia has the potential to provide new insights into the pathophysiology of the illness as well as to reveal novel targets for the disabling cognitive deficits seen in the illness.

In addition to changes in resting state connectivity, the cognitive functions associated with elements of the DMN have been shown to be altered in patients. Specifically, Liemburg et al. (2012) demonstrated that the degree of connectivity within the DMN in patients correlated with atypically reduced self-reflective awareness. Other groups have found reduced activation in the PCC while patients performed self-reflective tasks, when compared to healthy participants (van der Meer et al., 2013). Alternatively, it is possible that attention lapses in patients are a result of reduced activations in the task-positive regions (Anticevic et al., 2013), perhaps resulting in hyperactivation of the DMN as a consequence. Such a deficit would suggest that attention lapses may be related to dysfunction in same frontal-cingulate-parietal circuitry underlying the well characterized cognitive control deficits seen in the illness (Lesh et al., 2011).

We predict, based on previous research in healthy participants, that the hyperactivity within the DMN will result in an increased level of attention lapsing in patients with schizophrenia. However, little research has been done regarding attention lapses in these patients. It may be the case that lapses in patients occur due both to DMN intrusions and failures to activate task-positive regions.

We endeavored to distinguish between these possibilities using the AX-Continuous Performance Task (AX-CPT; **Figure 1**). We first identified the neural correlates of attention lapses in this task, and investigated the connectivity within and between the task positive and DMNs in healthy participants. We repeated these analyses on data gathered from patients with schizophrenia. We were then able to compare the effect of attention lapsing between these two groups.

**FIGURE 1:**
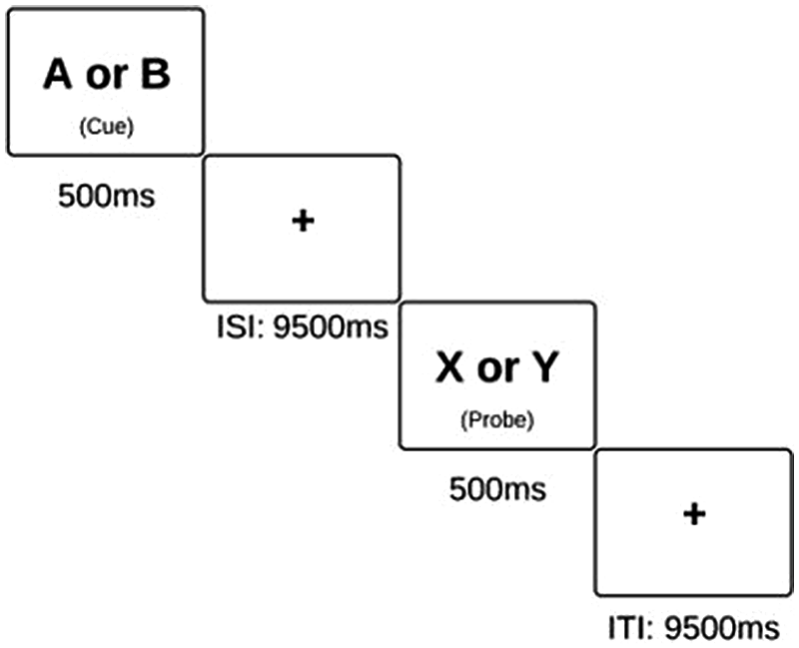
**Schematic of the AX-CPT task**. Subjects are instructed to press the “non-target” button in response to all cues and probes, excluding an X probe which was preceded by an A cue. On these X probes, subjects are instructed to press the “target” button.

## Materials and Methods

### Participants

All participant data was extracted from a previously published dataset of first episode (FE) schizophrenia patients (*n* = 32) and demographically matched comparison subjects (*n* = 23) who had performed the AX-CPT (Lesh et al., 2015). Participants were included in the present analysis if that had five or more AX/BY lapses. This study was approved by the institutional review board of University of California, Davis. Subjects provided written informed consent and were compensated for their participation in the study.

First Episode Psychosis patients, aged 18–35 were recruited from the EDAPT (Early Diagnosis and Preventive Treatment) Clinic in the Department of Psychiatry at UC Davis Medical Center and other sources as needed in Sacramento and Davis, CA, USA. Diagnosis was based upon the SCID and chart review and confirmed at consensus diagnostic conference. Patients were assessed using the Scale for the Assessment of Negative Symptoms, the Scale for the Assessment of Positive Symptoms, and the Brief Psychiatric Rating Scale (**Table 1**) (Andreasen, 1981, 1984; Lukoff et al., 1986). Healthy participants were recruited from the same communities as patients, through fliers and community service announcements. Exclusion criteria included IQ less than 70, history of neurological disorder, ECT, seizure disorder, significant head trauma or substance abuse or dependence within the previous 6 months. Subjects had their first onset of psychotic symptoms no more than 1 year prior to scanning. At the time of scanning, six of the subjects were unmedicated. The remaining 26 subjects were being treated predominantly with atypical antipsychotics. Further details can be found in Supplementary Table S1. Healthy participants were administered a urine drug screen at the initial assessment, which detects amphetamines, barbiturates, benzodiazepines, cocaine, alcohol, opiates, PCP, and THC. Positive results resulted in exclusion from the study. Healthy participants who have a neurological illness, history of head trauma leading to unconsciousness, or total score less on WAIS-R than 70 were also excluded. Only subjects with (corrected) vision of 20/30 or better were studied. Subjects were excluded if they have any contraindications to MRI. Groups were compared in terms of age, gender, IQ, and years of parental education (as an index of parental SES). Groups differed significantly in terms of age and gender. However, neither age nor IQ was a significant predictor on lapse numbers or beta values for any of the regions studied, as assessed using linear regression (*F* > 0.05 in all cases). Additionally, dividing the groups by gender did not impact either lapse numbers or beta values significantly (Chi-squared > 0.05 in all cases). As would be expected patients were impaired compared to controls on IQ as measured using the WASI (Wechsler, 1999).

**Table 1:** Patient Demographics.

	HC	SZ	Significance (*p*-value)
Age	21.3	24.7	*p* < 0.005
Gender	12 male, 11 female	25 male, 7 female	*p* < 0.05
IQ (WASI score)	110.08	99.46	*p* < 0.05
Parental years of education	15.14	14.81	N.S.
AX Lapses (sd)	9.45 (4.12)	9.71 (6.53)	N.S
BY Lapses (sd)	0.318 (0.56)	0.65 (0.90)	N.S.
Mean SANS score (sd)	-	2.18 (0.91)	-
Mean SAPS score (sd)	-	1.61 (0.97)	-
Mean BPRS score (sd)	-	42.5 (11.81)	-

### AX-Continuous Performance Task

This task was presented using E-Prime software in both behavioral conditions (using a laptop) and scanning conditions (in which the display is projected onto a screen). The AX-CPT consists of 4 blocks of 40 trials each. Each trial lasted a total of 14 s, and the entire task took 40 minutes to complete. Within each block, the participants are presented with pairs of letters in sequential order, and they are instructed to respond to the presentation of each letter with a button press. The first letter is a “cue” while the second is a “probe”. Specifically, they are instructed to respond with an answer of “non-target” to every letter except an “X” probe, which has been preceded by an “A” cue. AX trials make up 70% of all trials, and thus, a prepotent tendency to routinely respond to probes in the affirmative is established. AY trials compose 10% of the task, BX trials compose 12.5% of the task, and BY trials make up the remaining 7.5%. Failure to inhibit the tendency to respond “target” on an AY probe or on a BX probe are measures of cognitive control, as presented in previous studies (MacDonald and Carter, 2003). However, participants generally have a high level of accuracy on AX and BY trials. This is likely due to the fact that in AX trials, there is nothing in the task environment which should counteract this established tendency to respond to the X as a target. On BY trials, neither the cue nor the probe should prompt a response of “target.” Thus, in conditions in which the participant answers incorrectly on an AX or BY trial, we postulate that this was due to an attention lapse.

### Neuroimaging Data Acquisition

Using a 1.5 Tesla scanner, a localizer scan was run, followed by a 1.5 mm × 1.5 mm × 1.5 mm high-resolution T1 image using the MPRAGE sequence. The final scan was a T2^∗^-weighted gradient-echo EPI pulse sequence (TR = 2 s, TE = 40 ms, FA = 90, FOV = 22 cm, matrix: 64 × 64, voxel size: 3.4 mm isotropic). 288 volumes were acquired during each EPI sequence, which coincided with each task block.

### fMRI Preprocessing and Analysis

Preprocessing was performed using standard methods in SPM8 (Ashburner et al., 2013). This includes slice timing correction and motion correction, co-registration to the median image, and spatial normalization to a template of all functional images. Images are spatially smoothed with a Gaussian kernel (8 mm FWHM, isotropic).

SPM8 was used to implement general linear modeling (GLM) on an individual-subject basis. Each event was modeled by convolving a vector of expected neural activity with a hemodynamic response function. This vector was generated using in-house Matlab scripts which denoted the cue onset of AX or BY lapse trials, and AX or BY correct trials. All other trial types were also modeled in the GLM but not included in analysis. Nuisance variables were included to regress out motion-related signal changes. We set a maximum movement threshold of less than 1 voxel (3.4 mm) in a single dimension per block. The data is high-pass filtered to remove the influence of temporal autocorrelation. We determined the least-squares solution of the GLM in order to extract regression coefficients associated with each covariate of interest. Parametric maps were computed for each subject using the resulting t-statistics associated with linear combinations of covariates, in order to assess whether these combinations are significantly different from zero.

### Whole-Brain Analyses

In order to determine where brain activity was affected by lapsing, we performed multiple second-level analyses using a random effects analysis. Contrast images were created by calculating linear combinations of the single-subject parametric maps. These contrast images were subjected to a second-level *t*-test against a null value of zero. Separate one-sample *t*-tests were computed for healthy participants and patients for within-group analyses. We first contrasted AX/BY lapses with AX/BY correct trials in healthy participants. We repeated this contrast in patients with schizophrenia. We then contrasted the results from these two analyses, in order to determine whether the effect of lapses was significantly different between groups. All results were corrected for multiple comparisons using FWE correction at the cluster level, *p* < 0.05, with a voxel-wise threshold of *p* < 0.01.

### Region of Interest Selection

Connectivity analysis focused on six *a priori* regions of interest (ROIs) selected on the basis of being maximally activated/deactivated during a study of externally cued working memory (Fox et al., 2005). These are summarized in **Table 2**. In the DMN, these regions include the left and right vmPFC, and the left and right PCC. We confined our connectivity analysis of the frontal-parietal control network (FPCN) to the left and right dlPFC, as previous research has suggested that these regions may be particularly associated with attention lapses (Dixon et al., 2014). Finally, following our analysis of activation in patients, we added an additional 5 mm sphere in the left and right IPS ROI, again using coordinates from the study by Fox et al. (2005). From each set of coordinates, a 5 mm sphere was generated, and the time course of BOLD signal was used for functional connectivity analysis. In order to remove noise from the connectivity data, a CSF ROI was generated using the CompCor method of automated segmentation (Behzadi et al., 2007). The time course of this ROI was then used as a noise covariate.

**Table 2:** Regions of interest (All coordinates in MNI).

	Left	Right
Posterior cingulate cortex (PCC)	0, -33, 40 (bilateral)	0, -33, 40 (bilateral)
Dorsolateral prefrontal cortex (dlPFC)	-42, 45, 21	42,47,15
Ventromedial prefrontal cortex (vmPFC)	-4, 58, 2	2, 61, 13
IPL (reduced in SZ)	-42, -30, 50	53, -32, 56

### Functional Connectivity Analysis

We used the Conn toolbox for Matlab, as described in (Whitfield-Gabrieli and Nieto-Castanon, 2012). Using this toolbox, we were able to select the ROIs as seeds for seed-to-voxel connectivity analysis. These were conducted on individual participants for lapse and correct trial conditions, for each ROI listed in **Table 2**. These were entered into second-level analyses, in which statistical parametric maps indicating the level of connectivity during lapses were contrasted with maps indicating the level of connectivity during correct trials. Separate analyses were carried out for healthy participants and patients. We then conducted a between-group comparison in which we contrasted the maps resulting from the previous analysis between patients and controls for each seed. These results were FWE corrected for multiple comparisons, *p* < 0.05 at the cluster-level, with a voxel-wise threshold of *p* < 0.01.

## Results

### Within-Group Univariate Contrasts

**FIGURE 2:**
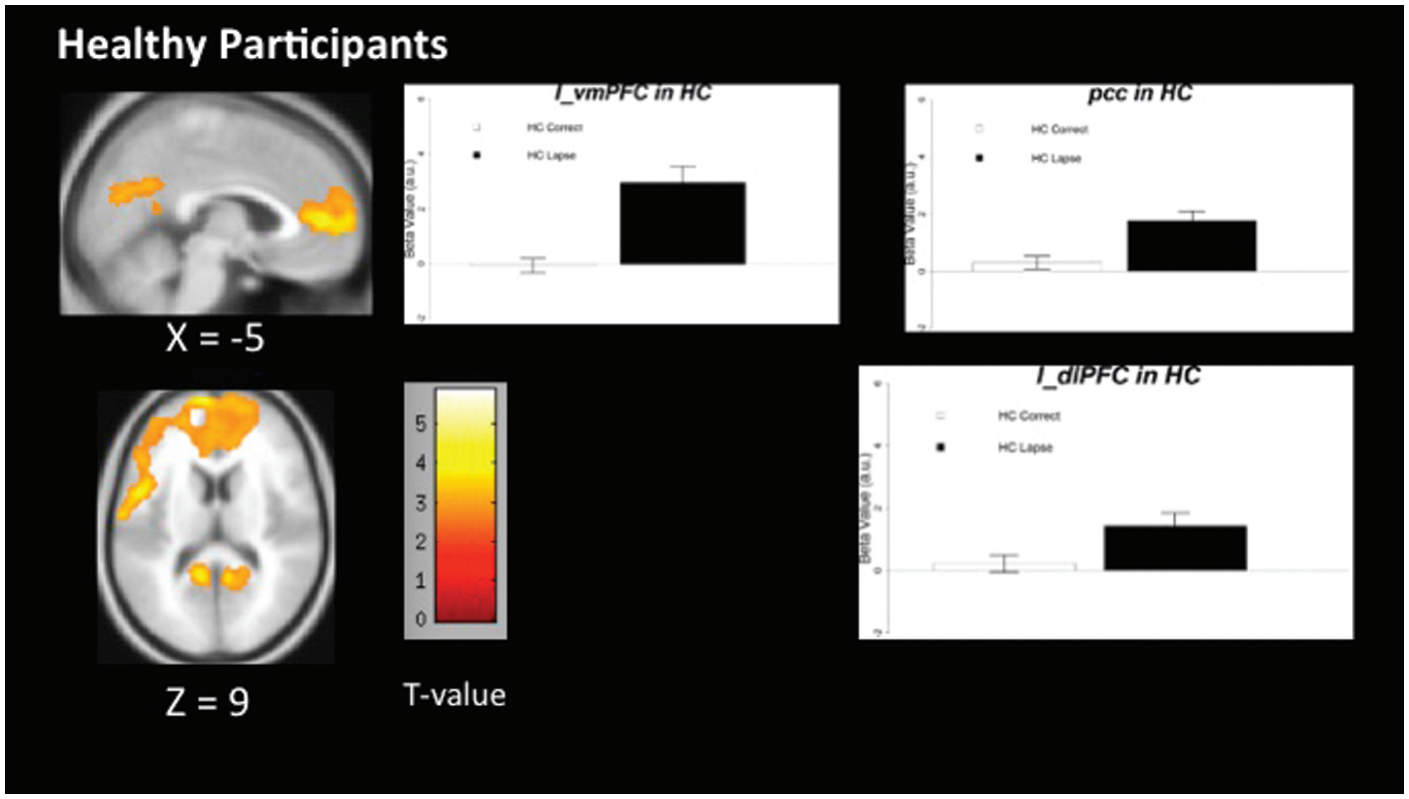
**Regions showing increased BOLD signal during lapses compared to correct trials in healthy participants**. Brain regions showing significant difference comparing AX lapses and AX correct trials. Cluster-level FWE corrected at *p* < 0.05, voxel-wise threshold *p* < 0.01. No significant reductions in BOLD signal were observed for healthy controls. Beta values are shown to the right of each figure, indicating beta estimates during correct trials and lapses.

**FIGURE 3:**
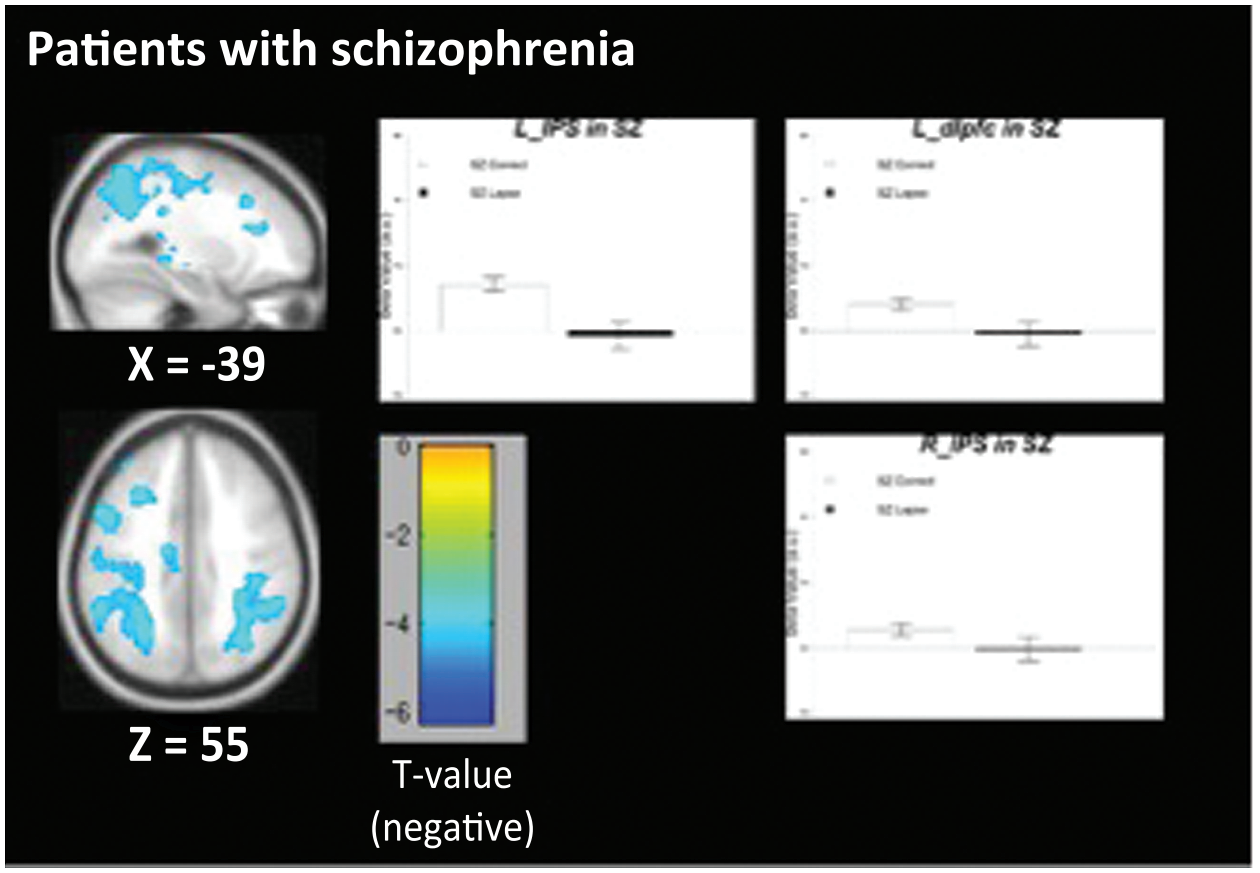
**Regions showing decreased BOLD signal during lapse trials compared to correct trials in patients with schizophrenia**. Beta values for right intraparietal sulcus (IPS), left IPS, and left dorsolateral prefrontal cortex (dlPFC) are shown. Error bars are ±SEM. Scale bar shows *t*-value for each voxel. Cluster-level FWE corrected at *p* < 0.05, voxel-wise threshold *p* < 0.01.

The pattern of activation observed in healthy participants (**Figure 2**) replicates findings in previous studies in which the PCC, vmPFC, and left dlPFC are active (see **Table 3** for coordinates and peaks of activation; Christoff et al., 2009). The results from patients (**Figure 3**) indicate a reduced level of activation in the left dlPFC, as well as the left IPS and right IPS during lapses. Notably, no change in DMN regions was observed in patients during lapses.

**Table 3:** Univariate results (all significant clusters above *p* < 0.05, FWE cluster corrected).

Region name	Peak MNI coordinates	Peak *t*-value
**In healthy participants**
Medial frontal gyrus	2, 46, 8	5.88
Medial frontal gyrus	-10, 66, 6	5.86
Superior frontal gyrus	-4, 58, 2	4.23
Precuneus	-14, -74, 26	5.10
Posterior cingulate	-10, -52, 14	4.56
Posterior cingulate	8, -52, 14	4.11
**In patients with schizophrenia**
Left inferior parietal lobule	-44, -38, 50	-6.21
Postcentral gyrus	-42, -30, 50	-5.47
Postcentral gyrus	-44, -20, 56	-5.34
	-48, 10, 42	-5.15
Middle frontal gyrus	-48, 10, 42	-5.15
Middle frontal gyrus	-30, 22, 38	-4.47
Sub-gyral left frontal white matter	-24, 26, 20	-3.93
Lateral Precuneus	34, -68, 38	-4.37
Sub-gyral right parietal white matter	30, -42, 40	-4.35
Right Superior Parietal Lobule	34, -52, 54	-0.02
**Between groups (HC-SZ)**
Middle frontal gyrus	-48, 10, 34	5.19
Middle frontal gyrus	-38, 40, 18	4.72
Superior frontal gyrus	-12, 64, 2	4.65
Posterior cingulate	-18, -50, 14	4.04

### Between-Group Contrasts

**FIGURE 4:**
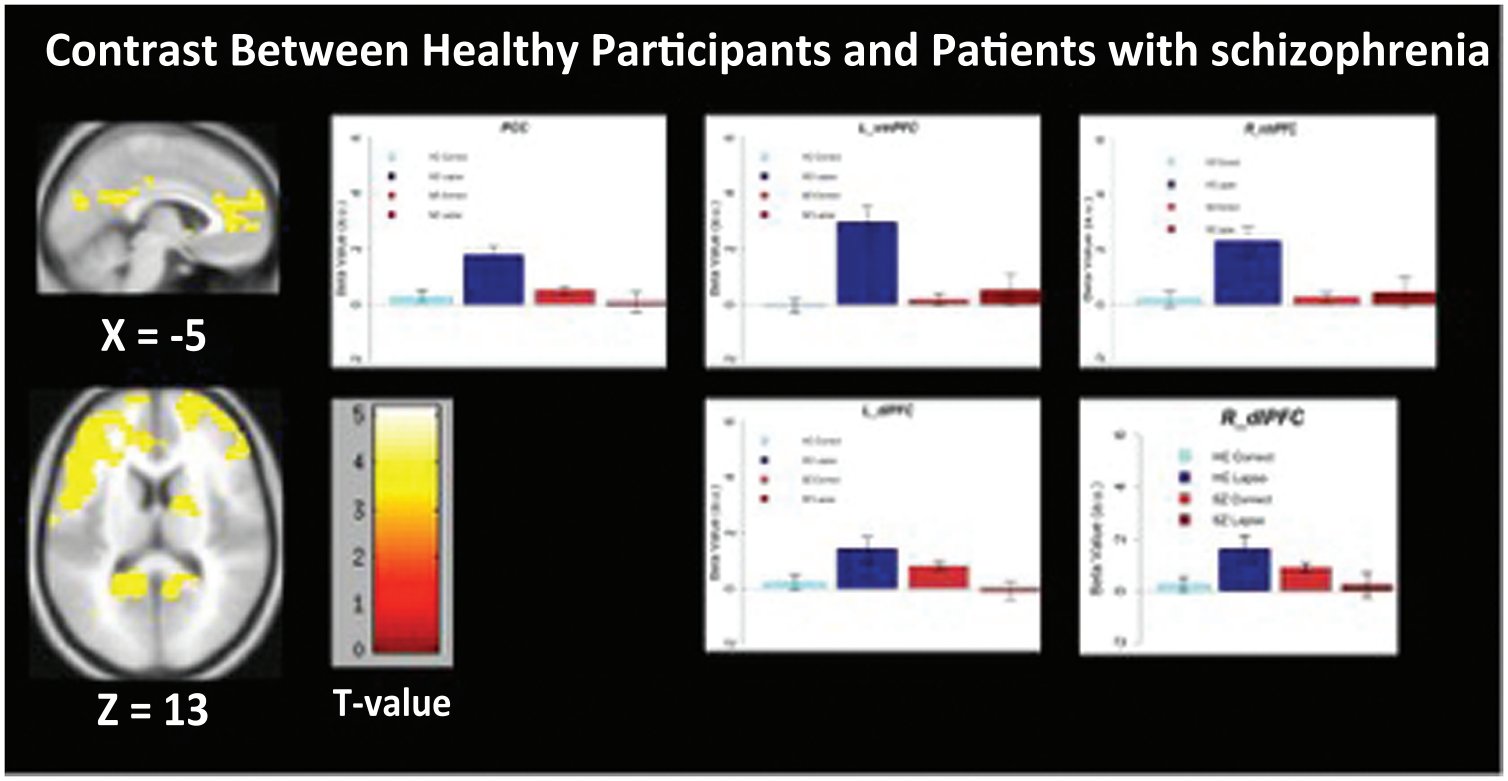
**Regions showing significantly greater activation in healthy participants compared to patients with schizophrenia**. Beta values for right mPFC, posterior cingulate cortex (PCC), and left dlPFC are shown. Error bars are ±SEM. Scale bar shows *t*-value for each voxel. Voxel-wise threshold *p* < 0.01, FWE-corrected at the cluster-level, *p* < 0.05.

We observed significant between-group differences in the lapse trials – correct trials contrast in both DMN and FPCN regions (**Figure 4**). Specifically, significantly increased signal was observed in left and right dlPFC (*p <* 0.05 FWE-corrected, MNI = -48, 10, 34) as well as left and right vmPFC (*p* < 0.05 FWE-corrected, MNI = -12, 64, 2/) and PCC/Precuneus (*p <* 0.05 FWE-corrected, MNI = -2, -60, 26) in controls rather than in patients. No regions were found to be more active in patients than controls during lapses.

### Functional Connectivity in Healthy Participants

**FIGURE 5:**
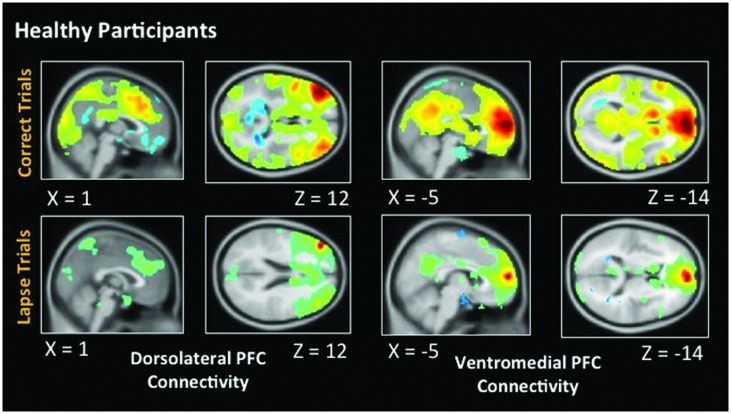
**Connectivity results for healthy participants**. Positive correlations are shown in hot colors. Anticorrelations are shown in cool colors. Dorsolateral PFC indicates voxels which significantly correlated with a 5 mm seed located at (-42, 45, 21). Ventromedial PFC indicates voxels which significantly correlated with a 5 mm seed located at (2, 61, 13). Voxel-wise threshold *p* < 0.01, FWE corrected *p* < 0.05 at the cluster level. Right-sided seeds yielded similar results.

**Figure 5** indicates voxels in which the voxel time course covaried significantly with a 5 mm spherical seed. The top of the figure shows connectivity during correct trials. Bilaterally, the dlPFC is functionally connected to a statistically significant degree with task-positive regions including the IPS, as well as visual regions. Anticorrelations between dlPFC and DMN regions are also evident. During lapse trials, this anticorrelation with DMN regions is no longer apparent, and functional connectivity with visual regions is no longer above statistical thresholds. In addition, the vmPFC is significantly connected with other DMN regions during correct trials. This level of connectivity was not observed during lapses in healthy participants. Furthermore, dlPFC connectivity with visual cortex was not observed during lapses.

### Functional Connectivity in Patients with Schizophrenia

**FIGURE 6:**
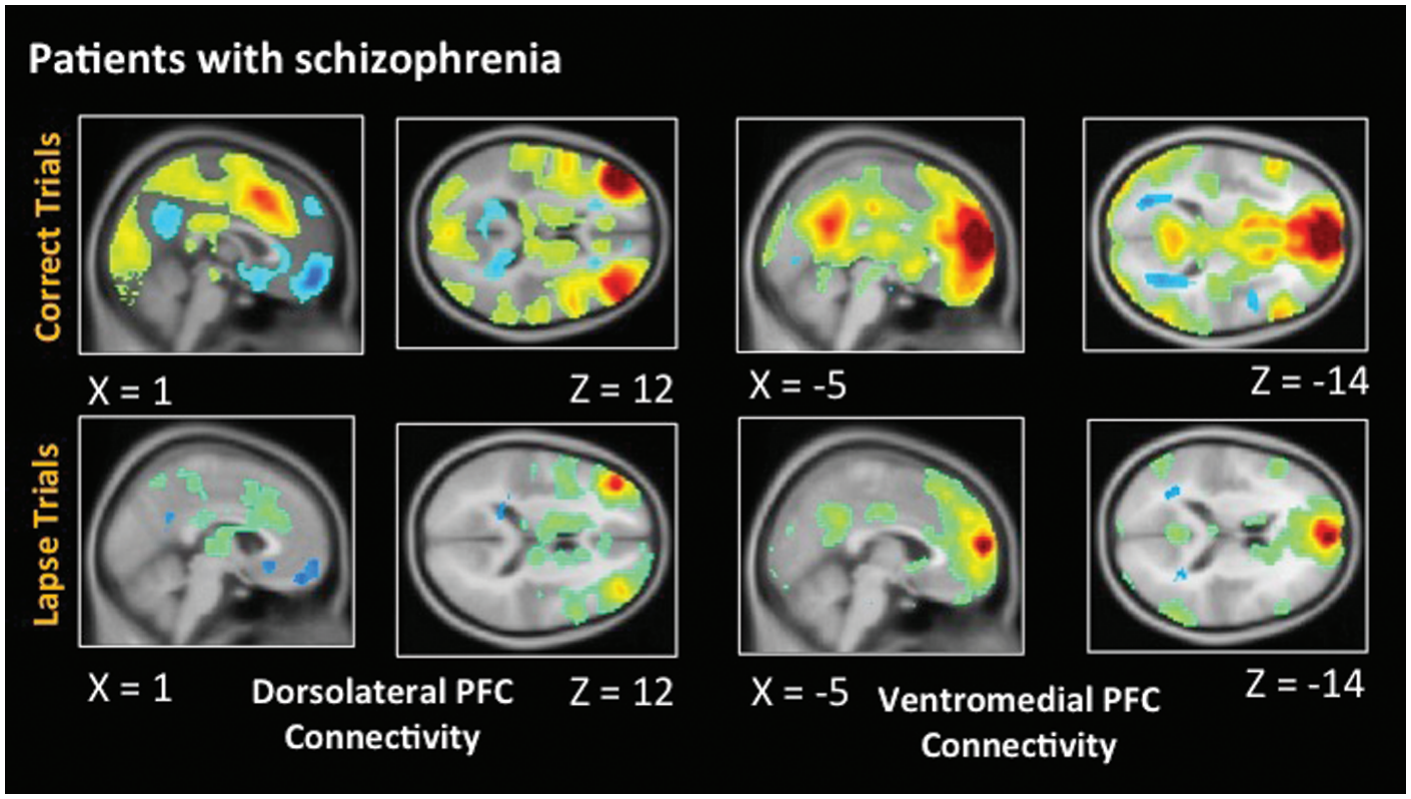
**Connectivity results for patients with schizophrenia**. Positive correlations are shown in hot colors. Anticorrelations are shown in cool colors. Dorsolateral PFC indicates voxels which significantly correlated with a 5 mm seed located at (-42, 45, 21). Ventromedial PFC indicates voxels which significantly correlated with a 5 mm seed located at (2, 61, 13). Height threshold *p* < 0.01, FWE corrected *p* < 0.05 at the cluster level.

As in healthy participants, we observed statistically significant functional connectivity between the dlPFC and other task positive regions during correct AX trials. Significant anticorrelation with the posterior cingulate can also be observed (**Figure 6**). During lapse trials, significant connectivity within FPCN regions is maintained, but this anticorrelation with DMN regions is no longer evident. As in healthy participants, connectivity between the dlPFC and visual regions is no longer statistically significant during lapses. The vmPFC is significantly functionally connected with DMN regions during both correct and lapse trials. Furthermore, during lapses in patients, significantly greater functional connectivity between the right IPL and DMN regions, including the left vmPFC and PCC, was observed, relative to correct trials. The left IPL, meanwhile, is connected to a significantly greater degree to bilateral dlPFC, as well as the right IPL.

### Between-Group Functional Connectivity

**FIGURE 7:**
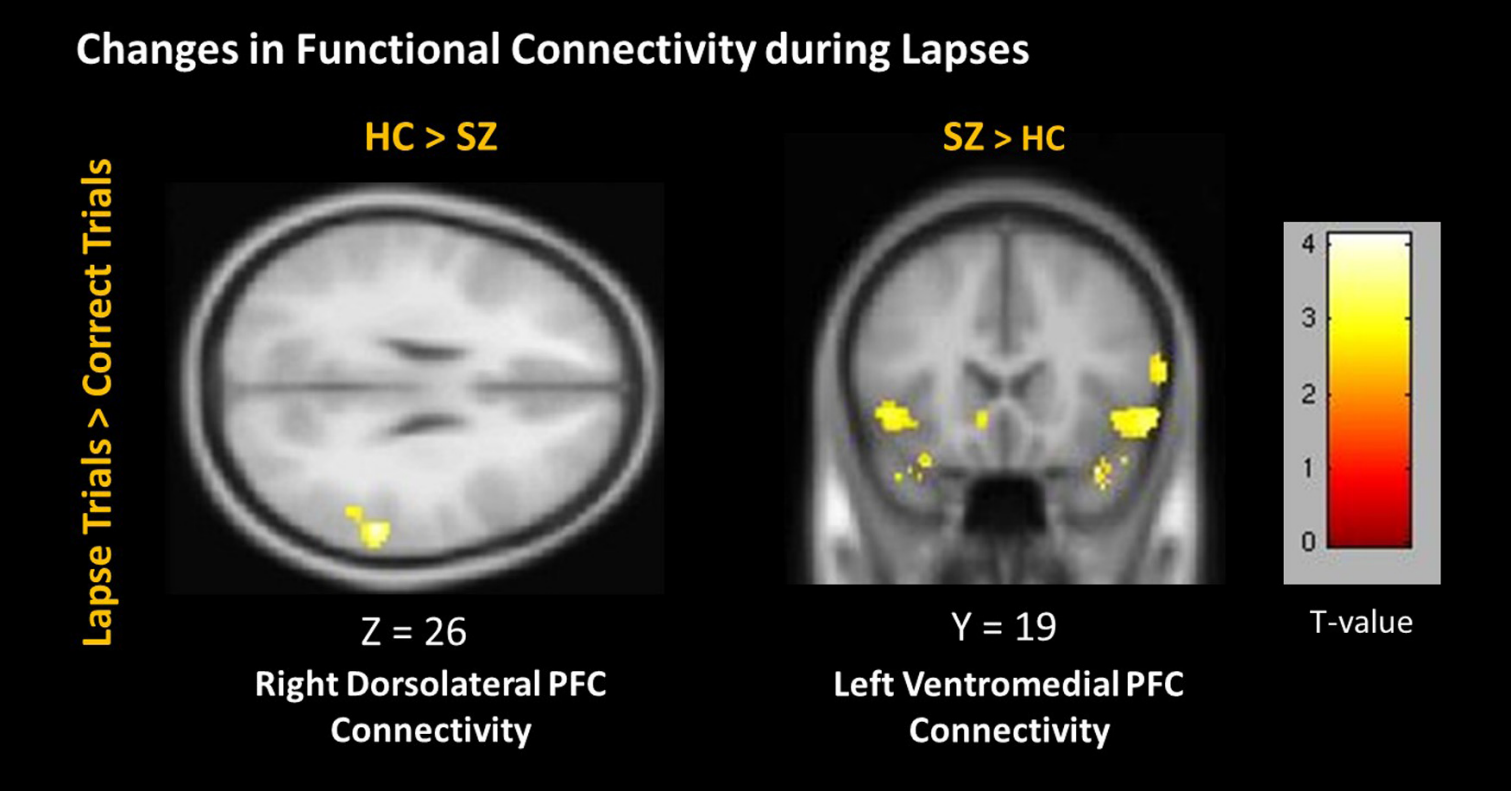
**Changes in connectivity during lapses between participant groups**. Positive correlations are shown in hot colors. Anticorrelations are shown in cool colors. Dorsolateral PFC Connectivity indicates voxels which significantly correlated with a 5 mm seed located at (-42, 45, 21). Ventromedial PFC Connectivity indicates voxels which significantly correlated with a 5 mm seed located at (2, 61, 13). Contralateral seeds yielded no significant results. Voxel-wise threshold *p* < 0.01, FWE corrected *p* < 0.05 at the cluster level.

During lapses, right dlPFC connectivity with right IPL was observed to significantly greater degree in controls than in patients (**Figure 7**). Left vmPFC connectivity with bilateral inferior frontal gyrus (IFG) was observed to a significantly greater degree in patients than in controls. No significant differences between groups were observed using the left dlPFC seed, the right vmPFC seed, left or right IPL seeds, or the PCC seed.

## Discussion

We observed distinct patterns of activation and connectivity associated with attention lapses in healthy participants and patients with schizophrenia, rather than the reduced DMN suppression we expected in patients. These results are summarized in **Table 4**. In healthy participants, we observed greater BOLD signal in DMN regions during lapses, when contrasted with correct trials. In the patient group, we found that lapses were associated with significant decreases in activation in FPCN regions, rather than the DMN hyperactivation we expected. We compared the effect of lapses between these two groups, and observed that healthy participants displayed significantly greater activation in FPCN and DMN regions, during lapses, compared to patients. In terms of connectivity, lapses were associated with greater connectivity between dlPFC and IPL in healthy participants, relative to patients. This is similar to previous studies reporting increased connectivity between FPCN regions during mind wandering (Christoff, 2012). Furthermore, patients showed significantly more connectivity between vmPFC and IFG, during lapses. This connectivity between DMN and FPCN regions is similar to previous findings using resting state fMRI, in which these networks were shown to be less segmented in patients (Woodward et al., 2011). Furthermore, Anticevic et al. (2013) have previously shown the IFG to be activated less in patients during the maintenance phase of working memory tasks, compared to controls. It may be the case that enhanced connectivity between the IFG and the vmPFC during lapse trials is responsible for disrupted activation during other focused tasks. Together, these results suggest that the neural systems underlying attention lapses in patients with schizophrenia are qualitatively different from those seen in healthy participants.

**Table 4:** Schematic summary of lapse > correct results.

Result	Effect of lapses in healthy participants	Effect of lapses in patients with schizophrenia	Significant differences between groups
Frontal-parietal control network (FPCN) activation	Increased	Decreased	Con > Schiz
Default mode network (DMN) activation	Increased	-	Con > Schiz
dIPFC-visual connectivity	Decreased	Decreased	Con = Schiz
dIPFC-IPL connectivity	Increased	-	Con > Schiz
FPCN-DMN anticorrelation	Decreased	-	Con > Schiz

### Distinct Patterns of DMN Activation in Healthy Participants and Schizophrenia Patients

Between-group comparisons indicate significantly different patterns of activation in both FPCN and DMN regions during lapses in healthy participants and schizophrenia patients. The directionality of these findings can be determined by examining the within-group contrasts in **Figures 2** and **3**. The findings in healthy participants resemble previous research regarding fluctuations in attention, in which several regions of the DMN present greater beta values during lapsing (Li et al., 2007; Mason et al., 2007; Sonuga-Barke and Castellanos, 2007). The within-group contrast in patients showed no change in the beta values of DMN regions, but a reduction in FPCN regions. This draws comparison with the findings of Weissman et al. (2006), in which it was reported that FPCN regions were less active prior to lapses in healthy participants. Importantly, that study showed a transient reduction in FPCN activity at the beginning of a lapse, while these results indicate that this reduction in activity is maintained during the lapse in patients. Further research is required in order to determine the cognitive implications of failures to maintain activation in the FPCN, but this could represent a loss of context or task set on the part of patients engaged in the task, which has been widely reported in previous studies of cognitive control in schizophrenia (Lesh et al., 2011).

### Patterns of Functional Connectivity in Healthy Participants and Schizophrenia Patients

Functional connectivity further suggested that the neural correlates underlying lapses are fundamentally different in healthy participants and patients. The between-groups contrast identified significantly greater connectivity between dlPFC and IPS in healthy subjects during lapses, relative to patients. This is consistent with previous studies which identified a greater degree of FPCN connectivity during mind wandering (Christoff, 2012). Patients, in comparison to controls, did show enhanced connectivity between DMN and FPCN regions, specifically the vmPFC and IFG. It is possible that this increased connectivity between FPCN and DMN networks may disrupt the functioning of the FPCN in patients, although further research is required.

### Relationship between the DMN and FPCN

It has previously been hypothesized that an intrinsic anticorrelation exists between these networks (Fox et al., 2005) and that competition or interference between them is a basis for impaired performance during periods of increased DMN activity. However, recent research suggests that DMN activation is not necessarily accompanied by FPCN deactivation, and vice versa. These networks have been observed to be strongly anticorrelated during resting state (Kelly et al., 2008), and our results indicate this anticorrelation is maintained during effective task performance. However, our results also suggest that under certain conditions (lapses) this anticorrelation is not stable and therefore not an intrinsic property of the brain’s architecture. Instead our results suggest that the nature of the relationship between these networks during task performance is actually quite dynamic with a loss of anticorrelation being associated with disruptions in task behavior. This is consistent with previous research suggesting that this anticorrelation is reduced during periods of reduced performance (Christoff et al., 2009). Precisely why this is the case remains unclear, though it has been hypothesized that internal stimuli ‘hijack’ the metacognitive functions which are required for effective task performance (Christoff et al., 2009; Dixon et al., 2014). It is alternatively possible that increased activation of the FPCN during attention lapses reflects an attempt to overcome the attention lapse, and reorient to the external stimulus (Weissman et al., 2006). Our activation and connectivity results in healthy participants are consistent with a view that in the context of an external cognitive control task like the AX CPT, suppression of the DMN is important for effective task performance and hence for adaptive brain functioning.

### Limitations and Future Directions

This study has several limitations. First, the number of lapses per subject in this study is limited. Our primary aim in this study was to make between-group comparisons of brain activity during lapsing, which necessitated a trade-off between number of subjects per group and number of lapses per subject. Future studies might consider extending the duration of the task, in order to capture a greater number of attention lapses. This would be particularly useful in increasing the spatial specificity of the clusters observed in contrasts, by permitting a higher voxel-wise threshold. This would also enable improved modeling of lapse trials in individual subjects, reducing differences in power between correct and lapse trials. Finally, atypical antipsychotics have previously been shown to influence connectivity within the DMN (Sambataro et al., 2010; Hadley et al., 2013). Lapse numbers as well as beta values for medicated and unmedicated patients can be found in the supplemental materials (Supplementary Figure S2).

## Conclusion

Our results replicate previous findings of an increase in BOLD signal in DMN regions preceding a lapse in healthy participants, when compared to correct trials. We also report a form of lapsing which has not been observed in healthy participants, but appears common in patients with schizophrenia, and which may correspond to failures to activate or maintain activation in task-positive regions. These results suggest that unlike healthy participants, attention lapses in patients with schizophrenia are more closely related to failures in the ability to engage or maintain activation of the FPCN previously associated with pervasive control deficits, rather than to the “mind wandering” that is associated with DMN intrusions in healthy participants (Minzenberg et al., 2009; Lesh et al., 2011). Future studies should further investigate the relationship between attention lapsing and performance deficits on perceptual and cognitive tasks in order to obtain a more comprehensive and definitive understanding of the neural underpinnings of cognitive disability in the illness.

## Author Contributions

RP analyzed data, drafted, and revised manuscript and figures. TS assisted in analysis. CS designed and oversaw experiment and contributed to the writing of the manuscript.

## Conflict of Interest Statement

The authors declare that the research was conducted in the absence of any commercial or financial relationships that could be construed as a potential conflict of interest.
