# Supplementary material for: Distinct neural correlates for attention lapses in patients with schizophrenia and healthy participants
Source: Front Hum Neurosci. 2015 Oct 6;9:502. doi: 10.3389/fnhum.2015.00502 (PMC4594500; doi:10.3389/fnhum.2015.00502)
Supplement: Supplementary file 1 [file Image_1.PDF]

Subject	Medications	Class
1	abilify (10mg)	Atypical antipsychotic
2	risperdal (4mg), cogentin (2mg)	Atypical antipsychotic, Benzodiazepine
3	zoloft (25 mg)	SSRI
4	abilify (5mg)	Atypical antipsychotic
5	Zyprexa (10-25mg), Risperidone(6mg), Atenolol(25mg), Benztropine(2mg), Lisinopril(10mg)	Atypical antipsychotic, Atypical antipsychotic, Beta blocker, Benzodiazepine
6	Abilify	Atypical antipsychotic
7	Olanzapine(10mg), Lorazepam(1mg)	Atypical antipsychotic, Benzodiazepine
8	Zyprexa(20mg)	Atypical antipsychotic
9	Lexapro	SSRI
10	Risperdal, Fluoxitine	Atypical antipsychotic, SSRI
11	Abilify, Prozac	Atypical antipsychotic,SSRI
12	Zoloft	SSRI
13	Zyprexa (15mg), Haldol (15mg), Divalproex Sodium (250mg)	Atypical antipsychotic, Typical antipsychotic, Mood stabilizer
14	Zyprexa , Cogentin	Atypical antipsychotic, Benzodiazepine
15	Abilify (15mg), Lithium (600mg)	Atypical antipsychotic, Mood stabilizer
16	Risperdal (2mg)	Atypical antipsychotic
17	Haldol (10mg), Cogenten	Typical antipsychotic, Benzodiazepine
18	Risperdal (2mg), Atenolol (50 mg)	Atypical antipsychotic, Beta blocker
19	Risperdal	Atypical antipsychotic
20	Abilify (10mg)	Atypical antipsychotic
21	Zyprexa (15mg), Risperdal (2mg), Lorazepam	Atypical antipsychotic,Atypical antipsychotic, Benzodiazepine
22	Seroquel (25mg)	Atypical antipsychotic
23	Abilify	Atypical antipsychotic
24	Risperdal (2mg)	Atypical antipsychotic
25	Geodon (240mg), Haldol (2mg), Cogentin (1mg), Sertraline (100mg), Depakote (1000mg)	Atypical antipsychotic, Typical antipsychotic, Benzodiazepine, SSRI, Mood stabilizer
26	Risperdol (3mg)	Atypical antipsychotic
27	unmedicated	-
28	unmedicated	-
29	unmedicated	-
30	unmedicated	-
31	unmedicated	-
32	unmedicated	-

Figure S1. Patient Prescriptions and Dosages
