# Supplementary figures and images for: Distinct neural correlates for attention lapses in patients with schizophrenia and healthy participants
Source: Front Hum Neurosci. 2015 Oct 6;9:502. doi: 10.3389/fnhum.2015.00502 (PMC4594500; doi:10.3389/fnhum.2015.00502)

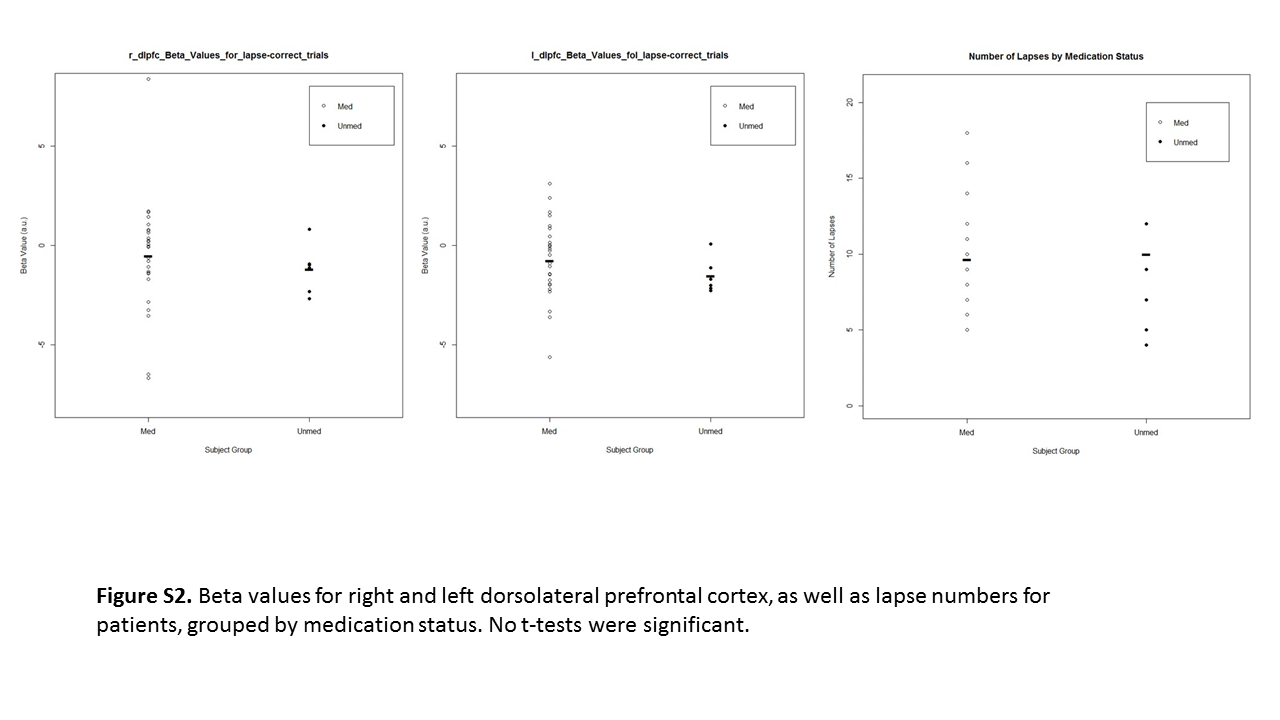

Supplement: Supplementary file 2 [file Image_2.TIF]
